# Health workers’ perceptions on where and how to integrate tobacco use cessation services into tuberculosis treatment; a qualitative exploratory study in Uganda

**DOI:** 10.1186/s12889-021-11502-4

**Published:** 2021-07-28

**Authors:** Elizeus Rutebemberwa, Kellen Nyamurungi, Surabhi Joshi, Yvonne Olando, Hadii M. Mamudu, Robert P. Pack

**Affiliations:** 1 Department of Health Policy, Planning and Management, Makerere University School of Public Health, Kampala, Uganda; 2 Centre for Tobacco Control in Africa, Kampala, Uganda; 3 Prevention of Non-communicable Diseases Division, World Health Organization, Geneva, Switzerland; 4 Department of Psychology, University of Nairobi, Nairobi, Kenya; 5 College of Public Health, East Tennessee State University, Johnson City, USA

**Keywords:** Tuberculosis (TB) treatment, Tobacco use cessation, Integration, Health workers

## Abstract

**Background:**

Tobacco use is associated with exacerbation of tuberculosis (TB) and poor TB treatment outcomes. Integrating tobacco use cessation within TB treatment could improve healing among TB patients. The aim was to explore perceptions of health workers on where and how to integrate tobacco use cessation services into TB treatment programs in Uganda.

**Methods:**

Between March and April 2019, nine focus group discussions (FGDs) and eight key informant interviews were conducted among health workers attending to patients with tuberculosis on a routine basis in nine facilities from the central, eastern, northern and western parts of Uganda. These facilities were high volume health centres, general hospitals and referral hospitals. The FGD sessions and interviews were tape recorded, transcribed verbatim and analysed using content analysis and the Chronic Care Model as a framework.

**Results:**

Respondents highlighted that just like TB prevention starts in the community and TB treatment goes beyond health facility stay, integration of tobacco cessation should be started when people are still healthy and extended to those who have been healed as they go back to communities. There was need to coordinate with different organizations like peers, the media and TB treatment supporters. TB patients needed regular follow up and self-management support for both TB and tobacco cessation. Patients needed to be empowered to know their condition and their caretakers needed to be involved. Effective referral between primary health facilities and specialist facilities was needed. Clinical information systems should identify relevant people for proactive care and follow up. In order to achieve effective integration, the health system needed to be strengthened especially health worker training and provision of more space in some of the facilities.

**Conclusions:**

Tobacco cessation activities should be provided in a continuum starting in the community before the TB patients get to hospital, during the patients’ interface with hospital treatment and be given in the community after TB patients have been discharged. This requires collaboration between those who carry out health education in communities, the TB treatment supporters and the health workers who treat patients in health facilities.

**Supplementary Information:**

The online version contains supplementary material available at 10.1186/s12889-021-11502-4.

## Background

Tuberculosis (TB) prevalence has been increasing in Uganda since the 1980s [1], accounting for 4% of total deaths in health facilities in 2018/2019 [2]. By 2018, TB prevalence nationwide was 200 (118–304) / 100,000 population [3], an increase from 58 (32–91) / 100,000 in 2013 [4]. Smoking has been cited as one of the risk factors contributing to this increase. The prevalence of smoking among patients with TB in Kampala was about 26% in 2015 [5].

In Uganda, TB treatment is under the TB and Leprosy Control Programme which is in the communicable disease control department while tobacco use cessation is under the docket of the Mental Health and Substance abuse programme in the non-communicable disease department. Tobacco use cessation and TB control programs are not linked [6]. At implementation level, whereas TB treatment is done in health facilities of all levels, tobacco use cessation is expected to be enforced by public health officers, environmental health officers, customs officers and local government officers who operate outside hospitals [7].

Tobacco use cessation activities can be offered in clinical or non-clinical settings. In clinical settings, these activities include recording smoking status of patients [8], giving routine advice to patients attending clinical care, offering intensive support with counselling to those who use tobacco and for some patients, administering nicotine replacement therapy. In non-clinical settings, they include educating the population on the dangers of tobacco use and second hand smoking [9].

The relationship between TB and tobacco use is well-established. A meta-analysis done in 2015 concluded that smoking was a risk factor for TB [10]. There is improved adherence to TB treatment with tobacco use cessation [11, 12]. Smoking has been associated with extensive lung disease [13], less treatment completion [14] and lower TB cure rates [8, 15]. Among patients with multi-drug resistant TB, smoking was associated with increased mortality [16]. In 2009, a study in China indicated an association between TB and tobacco smoking [17] which requires integration of interventions against TB with those against tobacco smoking.

Unfortunately, screening for smoking is not universal in all health facilities [18]. A study from South Africa in 2010 showed that many people seeking treatment in health facilities missed being screened for tobacco use [19]. A review of hospital records in Nigeria indicated poor documentation about tobacco use [20]. A literature review from low and middle-income countries (LMICs) indicated that physicians’ advice on quitting smoking was less among those who smoked than those who did not smoke [21].

Advice on tobacco use cessation has been shown to be effective. In a study involving 12 LMICs, physician’s advice increased the likelihood for increased utilization of smoking quit-lines, World Health Organization (WHO) - recommended tobacco use cessation medication, and cessation clinics [18]. Studies in India indicated that patients reduced tobacco use when they were asked about their smoking status and if found smoking were offered advice to quit smoking during their TB treatment [22, 23].

Health workers’ perspectives were sought because these health workers are key in the tobacco cessation exercise. Advice from health workers improves utilization of cessation assistance [18]. Health workers operate in both health facilities and communities where they carry out public health prevention and control activities. Understanding their perspectives would help in the design of interventions for tobacco cessation. Lack of integration between TB treatment work and tobacco cessation work in the communities was the missed opportunity which this study was trying to address. The results from this study would inform the integration agenda between the TB treatment and the Tobacco cessation programmes.

TB treatment is a chronic illness and tobacco cessation is a long term public health intervention. The Chronic Care Model (CCM) was chosen as the underlying model for this integration. The CCM has six main domains: community, health system, self-management support, delivery system design, decision support and clinical information systems [24]. It highlights adopting longitudinal, preventative, community-based and integrated approaches in the management for chronic illnesses. At community level, patients participate in community programs. At health system level, there is coordination within and across organizations; at delivery system design, there is regular follow up by the care system; for self-management support, there is empowering patients to manage their own conditions; at decision support, there is integration of specialist expertise and primary care; and for clinical information systems, there is identification of relevant populations for proactive care [25]. These are deemed critical in generating improved patient outcomes.

Lack of integration is a missed opportunity to support tobacco use cessation among patients with TB. Health workers who work with patients with TB are key stakeholders in tobacco use cessation activities. Physicians’ involvement in cessation services increases their uptake [18]. The objective of this study was to explore the perceptions of health workers on where and how tobacco use cessation services can be integrated into TB treatment in Uganda.

## Methods

### Study area

The study was conducted in nine health facilities: two from Kampala, which is the capital city; two from the eastern, three from the northern, and two from the western parts of the country. Three of the facilities were high volume health centres, two were general hospitals and four were regional referral hospitals. One general hospital was owned by a non-governmental organization (NGO) and the rest by the government. All these hospitals were offering primary and secondary level treatment. This is shown in Table 1.

**Table 1:** Characteristics of hospitals

Region	Regional Referral Hospitals	General Hospitals	Health Centre	Total
North	2	1	0	3
Kampala	0	0	2	2
Eastern	1	0	1	2
Western	1	1	0	2
**Total**	**4**	**2**	**3**	**9**

### Study design

This was a qualitative exploratory study conducted using focus group discussions (FGDs) and key informant interviews (KIIs). FGDs are useful in getting perceptions of certain groups over a particular issue [26]. During the FGD, interaction between respondents is used to explore their views and whether they coalesce on expressed opinions [27]. When respondents in KIIs are well informed, they provide an overall view of the community [28] and are less influenced by the presence of their peers [29].

### Study population

FGD respondents comprised of health workers working in TB clinics or in departments involved in the management of TB patients, such as out-patient departments, admission wards, laboratory, or dispensing section. They were purposively selected. They were mobilized by the head of department of the TB clinic through face-to-face discussions with heads of their respective departments. Department heads forwarded persons with experience in working with TB patients. Heads of departments for TB clinics were the key informant interviewees. There were a total of 81 respondents in FGDs with 27 male (33.3%) and 54 females (66.7%). Among the KIIs, there were 4 male (50%) and 4 females (50%).

### Data collection

Data was collected between March and April 2019. Eight research assistants (four male and four female) with at least 5 years of experience in qualitative research methods and a degree in social sciences were trained for 3 days on study objectives and ethical conduct in qualitative data collection. KII and FGD guides were developed for this study and had been approved in the proposal by the Institutional Review Board. The KII and FGD guides are attached as supplementary files 1 and 2.

There were nine FGDs having six to 13 respondents each and a total of 81 respondents. They included nurses, midwives, clinical officers, laboratory technologists, medical officers, counsellors, nursing assistants and data officers. None of them had implemented tobacco use cessation in their clinical practice because such integration is not government policy in Uganda. FGDs were conducted in a room of preference in facilities where respondents were working. Eight KIIs were also conducted in respondents’ preferred venues which were mostly their offices. FGDs and KIIs were conducted in English by a moderator and a note taker, 1 a female and the other a male. They were all audio recorded after securing consent. During the FGD or KII, the moderator and note taker kept summarizing key issues from the discussion to get confirmation from the respondent. FGDs took between 45 and 80 min while KIIs took between 25 and 40 min.

### Data management and analysis

FGDs and KIIs were transcribed verbatim by research assistants. Note takers’ notes and observations were inserted into the text. Midway through data collection, investigators reviewed the data to ensure key issues were coming out. Data was transferred into the Qualitative Data Analysis (QDA) Miner 2.0.6 (Provalis Research, Montreal, Quebec, Canada), a qualitative data analysis software. Content analysis was used to group similar responses [30]. Two investigators generated and agreed on the codes and any disagreement was resolved by mutual agreement. Meaningful units were used to generate codes which were later merged into themes. The chronic care model was used as a framework for analysis. Findings have been displayed using representative quotes. Consolidated criteria for reporting qualitative research (COREQ) reporting guidelines were used [31]. After the data analysis, it was noted that there was saturation already on the key themes. The results highlight the saturation areas.

## Results

Health workers highlighted all the six domains of the chronic care model as pertinent in the integration of tobacco cessation into TB treatment and control programs in addition to health system strengthening. 1) The integration was perceived to be needed in community programs; 2) Coordination with different organizations in the health system was needed; 3) There was need for regular follow up and self-management support; 4) Patients needed to be empowered to know their condition and their caretakers also be involved; 5) Integration of specialist with primary health care facilities; and 6) Instituting clinical information systems that would identify the relevant people for proactive care and follow up. 7) Respondents indicated that to achieve integration, the health system needed to be strengthened especially health worker training and provision of more space in some of the facilities.

1. Integration into community programs

Most of the respondents indicated that tobacco use cessation activities needed to be integrated into health promotion activities when sensitizing communities against TB.

*“The first thing is to create awareness in the community about the relationship between tobacco use and TB. When someone understands the benefits, one will stop using tobacco”* (KII 1 in-charge TB clinic).

2) Coordination with different organizations

The media was identified as an important partner in tobacco use cessation.

*“I feel the media can be used to sensitize people like on radio and newspapers so that people can get the information”* (FGD 9 Health workers).

Peers and community members are the other partners health workers saw as helpful in ensuring there is tobacco use cessation.

*“We should train peers who will be in the community. Peers will follow them up.”* (FGD 6 Health workers).

TB treatment requires having a trusted person whom the patient chooses to support him or her. This person gives drugs to the patient who takes them under observation. These people are called TB treatment supporters. TB treatment supporters were identified as possible partners in tobacco use cessation.

*“Treatment supporters are there to monitor these patients on treatment but also they can help to continuously encourage the patient on quitting tobacco smoking”* (FGD 9 Health workers).

“*… Patients have a habit of leaving the bed saying they want to walk around whereas they are going to smoke somewhere where they are not seen. The treatment supporter can inform the health worker that the patient has gone to smoke”* (FGD 4 Health workers).

3) Self-management support and regular follow up

Patients needed to be followed up after treatment so that they do not go back to tobacco use.

*“… it’s better to follow that person after the treatment for TB to see whether she went back to smoking or has remained without using tobacco”* (KII 7 in-charge TB clinic).

*“There should be follow-up of patients that have completed TB treatment on whether they have gone back to smoking or have completely stopped.”* (KII 8 in-charge TB clinic).

There was agreement across the respondents that there is lack of a robust investigation and follow up to monitor decrease in smoking.

*“… currently we cannot quantify how much tobacco someone consumes … we don’t have any investigation that is currently carried out at the health facility to see how much one has reduced. In TB, you do the Gene Xpert at the beginning,, then on follow-up at two months, you take off a sputum sample to see the progress of the treatment but regarding tobacco use, there is no quantification”* (FGD 3 Health workers).

Some respondents indicated that there was lack of Information Education Communication (IEC) materials and job aids to support self-management in tobacco use cessation.

*“I will suggest that the counselling can continue but also the Information Education Communication (IEC) materials like the posters for tobacco use cessation must be provided in public places so that after counselling somebody will look at the poster and say, ‘If this is the outcome, let me stop it’”* (FGD 4 Health workers).

*“… there is need for some job aids on tobacco use cessation because there is some other detailed information we are lacking and you can’t know everything. If there are those job aids, it can help us a lot”* (FGD 6 health workers).

4) Delivery system design and empowering patients

Most of the respondents indicated that tobacco cessation needs participation from the patients themselves. They need to be also supported by their caretakers.

*“… when the patient is educated, he will stop and that message is given to every patient who is started on treatment”* (FGD 5 Health workers).

5) Decision support and integration across levels of care

Respondents indicated that those who would have developed complications needed to be referred to specialized centres.

*“… for those who will have developed complications like cancer; we need to refer them to higher level facilities”* (FGD 3 Health workers).

6) Clinical information systems

Most respondents indicated that while at health facilities, smokers should be identified from other patients so that they can receive tobacco use cessation messages alongside their other treatment.

*“You identify these smokers so that you are able to give them key messages that will help them understand the relationship between TB and Tobacco use, what impact it has on the patient, on the patient’s treatment outcome, and on the community.”* (FGD 9 Health workers).

Documentation in facilities was identified as weak by majority of the key informants indicating that it did not record those who use tobacco. This made it difficult to follow up those smoking.

*“… the challenge could be that there is no proper documentation of whoever is a smoker … ..there is no way one can understand that this is a smoker and taking TB treatment. … a system should be built so that all data are captured and at the end of the day you know where to start from.”* (FGD 2 Health workers).

*“… of course we also ask about the smoking. You just do it verbally but you don’t document it anywhere. He may tell you that now I have reduced smoking but we don’t document”* (FGD 3 Health workers).

However, there were a few dissenting voices who indicated that the Ministry of Health tools were capturing the smokers. To them, it was an issue of data management where those who summarized the data were not giving the data on smokers.

*“Ministry of Health tools are already capturing it only that by the time the form reaches the Health Management Information System (HMIS) person to track that information, you find that that part was not filled.”* (FGD 3 Health workers).

7) Strengthening the health care system

Despite the strong support for the integration of tobacco cessation in TB treatment, the health care system needed to be strengthened. Majority of the respondents reported lack of skills for tobacco use cessation and having inadequate health workers on TB wards.

*“I may be having knowledge but we are working with other staffs who are not having the knowledge for tobacco use cessation yet they are part of the team. So they may not have knowledge that they require to implement the tobacco use cessation into TB treatment”* (KII 1 in-charge TB clinic).

*“Health workers are not enough to provide adequate services. For example, we don’t have a trained qualified counsellor to support TB patients. Sometimes a health worker can be alone at the out-patient and at the same time be on the ward to attend to patients”* (KII 2 in-charge TB clinic).

A minority of health workers however indicated that integration would not add on too much workload.

*“It doesn’t add any workload because we don’t get many people smoking. They are mostly men and most men don’t come to health facilities. The ladies who come mostly chew tobacco and that one is easily stopped once you advise them.”* (KII 8 in-charge TB clinic).

Some facilities especially the health facilities and general hospitals among the study sites indicated that they needed more space.

*“Space is not enough for health workers to do their work. Sometimes we utilize the small space for counselling services to provide TB treatment. Our dispensing room is also used by the staff as dressing room and meeting place”* (KII 2 in-charge TB clinic).

*“Ideally, space has been a challenge and it is still a challenge … at the moment the space where we are providing services is not adequate but I hope that in future we may have better space”* (KII 6 in-charge TB clinic).

However, some other facilities especially the bigger referral hospitals indicated that they had enough space depending on whether they had a designed building for specifically for TB or had small numbers of patients.

*“We have a whole unit that is built for handling TB management … we have space where we can manage them except if their number increases that is when we can face challenges”* (KII 1 in-charge TB clinic).

*“… we have enough space because patients can be two or three and also those who are diagnosed on TB treatment are not more than three a day, so that’s an affordable number which we can handle with the given resources”* (KII 7 in-charge TB clinic).

## Discussion

There are seven key findings on where and how tobacco use cessation services can be integrated into TB treatment. First, integration should go beyond health facilities to communities. Second, there is need for coordination across different organizations. Third, patients need to be followed up with support from the care takers. Fourth, patients need to be empowered to know their condition. Fifth, linkages need to be established between primary health care and specialized facilities. Sixth, those who use tobacco need to be identified in the system and be given pro-active follow-up. Lastly, challenges like inadequate numbers and skills in human resources and lack of space in health facilities need to be addressed.

Previous studies also stressed the importance of linking tobacco cessation to TB treatment and control at community level. A study in Hong Kong concluded that smoking cessation reduces secondary transmission and relapse of tuberculosis [32]. Tobacco use cessation needs to be integrated into the entire spectrum of TB health care continuum from prevention to post treatment management. Communities sometimes do not have proper knowledge about TB [33]. Other studies have also highlighted the coordination needed between tobacco treatment and other partners. Integration of tobacco use cessation into TB treatment involves TB treatment supporters, patient’s peers and the community. Treatment supporters are those people who support health workers in giving drugs to patients under the Community Based-Directly Observed Treatment Short course (CB-DOTS). A review of the CB-DOTS strategy was found to lead to improved TB treatment outcomes [34]. A study in Indonesia recommended integration of TB treatment and follow up with smoking cessation [35]. Empowering patients to know their condition has also been highlighted in other studies. As smoking is a risk factor for acquiring TB infection [36] integrating tobacco use cessation into TB prevention would address the incidence of TB. Healthy people should be sensitized about the relationship between tobacco use and TB. Even after TB treatment, tobacco use has been associated with reduced lung function [37]. Referral systems in the integration have been recommended as some conditions could be severe. For those who develop complications, there should be an opportunity for referral. A study in India highlighted inadequate referral to tobacco use cessation specialized centres [38]. The importance of documentation has been stressed. Documentation is not only to be initiated but also maintained. Some programs in India that initiated the integration later declined in documentation of tobacco users in TB treatment programs [22]. System strengthening has been mentioned as critical for this integration. Studies in South Africa noted a need for training and acquisition of other skills for the success of integration [39, 40].

For policy formulation and practice, health education for TB prevention and control needs to incorporate tobacco use cessation at community and health facility level. Health facilities need to be equipped with appropriately trained human resources in adequate numbers, have an information management system that can track those who use tobacco and avail the health workers with the needed IEC materials, job aids and other supplies. Referral within health service delivery is a critical factor in providing continuity in provision of services. Tailor made assessments for each facility need to be conducted for integration to be effectively done as different facilities have different capacities and resources to support additional activities. The program managers for TB treatment and control and tobacco cessation at central level like Ministries of Health need to align their programs so that there is harmonious integration at facility and community levels.

In addition to weak health systems that need to be strengthened in order to support the integration of tobacco cessation into TB treatment, this study highlights the use of peers and caretakers for TB patients to support the tobacco cessation. The role of these different groups in tobacco cessation could be explored further.

### Strengths and limitations of this study

The strength of the study was that all the respondents were health workers who interface with TB patients in their daily work and therefore could give informed opinions. The other strength was that sampled facilities included both urban and rural facilities hence experiences from both localities could be captured. The limitation was that the study was conducted in referral health facilities, general hospitals and high-volume health centres which may not be representative of all health facilities in the country. However, since most TB patients get treated in these high volume centres, changes in such centres could affect the highest number of patients and recommendations given by the health workers go beyond facilities to communities shared by both high level and lower level facilities.

## Conclusion

To health workers, the information system should identify smokers, counsel them on quitting tobacco use and follow them even beyond their stay at the health facility. For patients with complications of tobacco use, they need specialised treatment centres. Other stakeholders like treatment supporters, media and peers need to support tobacco use cessation. Tobacco use cessation in TB treatment and control integrates a community public health intervention implemented through public health laws and a chronic disease treated at health facilities. Integration should therefore not be restricted to health facilities but be linked to activities in communities. This could be done during prevention of TB and post treatment rehabilitation and management. Implementing such interventions may necessitate increasing the capacity and resources of some health facilities.

## Supplementary Information

**Additional file 1:.** Tool Key Informant Interview guide. Guide questions. Questions guiding the key informant interviews

**Additional file 2:.** Tool Focus Group Discussion Guide. Guide questions. Questions guiding the focus group discussions

## Data Availability

The data used for this paper has got many personal identifiers and places common in qualitative data. It can be got on reasonable request from the Principal Investigator of the study who is the first author on this paper.

## Abbreviations

CB-DOTS: Community Based Directly Observed Short course
CCM: Chronic Care Model
COREQ: Consolidated Criteria for Reporting Qualitative research
FGD: Focus Group Discussion
HMIS: Health Management Information System
IEC: Information Education Communication
KII: Key Informant Interview
LMICs: Low and Middle Income Countries
NAS: National Academy of Sciences
NGO: Non-Governmental Organization
NIH: National Institutes of Health
PEER: Partnership for Enhanced Engagement in Research
QDA: Qualitative Data Analysis
TB: Tuberculosis
USAID: United States Agency for International Development
WHO: World Health Organization
