# Supplementary material for: Health workers’ perceptions on where and how to integrate tobacco use cessation services into tuberculosis treatment; a qualitative exploratory study in Uganda
Source: BMC Public Health. 2021 Jul 28;21:1464. doi: 10.1186/s12889-021-11502-4 (PMC8317326; doi:10.1186/s12889-021-11502-4)
Supplement: Supplementary file 1 — Additional file 1:. Tool Key Informant Interview guide. Guide questions. Questions guiding the key informant interviews [file 12889_2021_11502_MOESM1_ESM.docx]

**TB- tobacco integration project**

**KII Guide for Health Workers;**

**Objective 1; Understanding the existing practice of TB- tobacco integration**

1. What is your position/title? How long have you been in this position at this facility/organization?
2. How is tobacco cessation integrated into TB treatment in this facility? (what it entails, how it is done)
3. Who is involved in the integration of tobacco cessation into TB treatment at this facility? (Probe for, adequacy of personnel; space for improvement?)
4. What is the existing capacity of this facility to integration of tobacco cessation and TB treatment? (Probe for human resource availability, medicines and supplies, space, skills)

**Objective 2; Establishing health workers knowledge on benefits of Tb – tobacco integration to their patients.**

1. In your opinion, what are the benefits of integrating tobacco cessation and TB treatment to the patient? (probe for access to TB services, cessation of tobacco use, cure rate)
2. What are the consequences if tobacco cessation is not integrated in TB treatment?

**Objectives 3; Assessing their perception on their role, knowledge and skills to do integration.**

1. What in your opinion is the role of health workers in integrating tobacco cessation activities in TB treatment? (probe for what they are doing and how)
2. In your view, do health workers have sufficient knowledge and skills to integrate tobacco cessation in TB treatment? (probe for the WHO 5 A of Ask, Advise, Assess, Assist and Arrange)
3. What are the challenges to integrating tobacco cessation activities into TB treatment? (*Probe for health worker-specific and facility-based challenges*)
4. What can be done to improve the integration of tobacco cessation into TB treatment clinics *(Probe for support for health workers and health facility?)*
