# Supplementary material for: Health workers’ perceptions on where and how to integrate tobacco use cessation services into tuberculosis treatment; a qualitative exploratory study in Uganda
Source: BMC Public Health. 2021 Jul 28;21:1464. doi: 10.1186/s12889-021-11502-4 (PMC8317326; doi:10.1186/s12889-021-11502-4)
Supplement: Supplementary file 2 — Additional file 2:. Tool Focus Group Discussion Guide. Guide questions. Questions guiding the focus group discussions [file 12889_2021_11502_MOESM2_ESM.doc]

**TB-Tobacco Integration Project**

**FGD guide for health workers in TB clinics**

**Demographic characteristics:**

| **SN** | **Sex** | **Cadre** | **Period** **of service** |
| --- | --- | --- | --- |
| 1 |  |  |  |
| 2 |  |  |  |
| 3 |  |  |  |
| 4 |  |  |  |
| 5 |  |  |  |
| 6 |  |  |  |
| 7 |  |  |  |
| 8 |  |  |  |
| 9 |  |  |  |
| 10 |  |  |  |
| 11 |  |  |  |

**Objective 1: Understanding the existing practice of TB-Tobacco integration**

1. Has any of you managed any person suffering from TB? Was any of those patients smoking or using tobacco in any form?
2. How are TB patients managed here? Do they get Tobacco cessation services too?
3. What are the existing practices in relation to tobacco cessation activities in TB treatment in your facility? (**probe** for; existence of service, what do the services entail, how is it done, by who, why)

**Objective 2: Establish health worker knowledge on benefits of TB-Tobacco integration to their patients**

1. What would be the benefits of integrating the tobacco cessation activities in TB treatment? Why should tobacco cessation be integrated in TB treatment?
2. Has anyone been trained in the five WHO As? *The 5As include (Ask, advise, assess, assist and arrange)?*

**Objective 3: Assessing perception on their role, knowledge and skills to do integration**

1. What do you think is the role of health workers in providing Tobacco related activities within TB treatment?
2. What is your opinion about health worker training in providing Tobacco related activities within TB treatment?
3. As health workers, how would you want to be supported to do Tobacco related activities within TB treatment?
4. How can health facilities be supported to offer tobacco cessation?

**Thank you very much for your time and views**
